# Molecular Integrative Analysis of the Inhibitory Effects of Dipeptides on Amyloid β Peptide 1–42 Polymerization

**DOI:** 10.3390/ijms24087673

**Published:** 2023-04-21

**Authors:** Nan Yuan, Lianmeng Ye, Yan Sun, Hao Wu, Zhengpan Xiao, Wanmeng Fu, Zuqian Chen, Yechun Pei, Yi Min, Dayong Wang

**Affiliations:** 1 Laboratory of Biopharmaceuticals and Molecular Pharmacology, School of Pharmaceutical Sciences, Hainan University, Haikou 570228, China; 2 One Health Cooperative Innovation Center, Hainan University, Haikou 570228, China; 3 Department of Biosciences, School of Life Sciences, Hainan University, Haikou 570228, China; 4 Key Laboratory of Tropical Biological Resources of the Ministry of China, Hainan University, Haikou 570228, China

**Keywords:** amyloid β peptide, aggregation, dipeptide inhibitors, molecular dynamic, pharmacology

## Abstract

The major pathological feature of Alzheimer’s disease (AD) is the aggregation of amyloid β peptide (Aβ) in the brain. Inhibition of Aβ_42_ aggregation may prevent the advancement of AD. This study employed molecular dynamics, molecular docking, electron microscopy, circular dichroism, staining of aggregated Aβ with ThT, cell viability, and flow cytometry for the detection of reactive oxygen species (ROS) and apoptosis. Aβ_42_ polymerizes into fibrils due to hydrophobic interactions to minimize free energy, adopting a β-strand structure and forming three hydrophobic areas. Eight dipeptides were screened by molecular docking from a structural database of 20 L-α-amino acids, and the docking was validated by molecular dynamics (MD) analysis of binding stability and interaction potential energy. Among the dipeptides, arginine dipeptide (RR) inhibited Aβ_42_ aggregation the most. The ThT assay and EM revealed that RR reduced Aβ_42_ aggregation, whereas the circular dichroism spectroscopy analysis showed a 62.8% decrease in β-sheet conformation and a 39.3% increase in random coiling of Aβ_42_ in the presence of RR. RR also significantly reduced the toxicity of Aβ_42_ secreted by SH-SY5Y cells, including cell death, ROS production, and apoptosis. The formation of three hydrophobic regions and polymerization of Aβ_42_ reduced the Gibbs free energy, and RR was the most effective dipeptide at interfering with polymerization.

## 1. Introduction

Alzheimer’s disease (AD) is a neurodegenerative disease that is the seventh greatest cause of death in the United States and the main cause of dementia in the elderly [1,2,3]. There is no known cause for the disease, which has an insidious onset and is difficult to detect in the early stages, worsens in the latter stages, and progresses extremely slowly. The clinical signs of AD include cognitive and memory decline, progressive loss of daily living abilities, and behavioral disorders culminating in death. An effective diagnostic tool or treatment is yet to be discovered [4,5,6]. Alzheimer’s disease is a widespread disease among the elderly. The number of sufferers is gradually increasing as the world’s population ages. The global population of people with Alzheimer’s disease doubles every 20 years. By 2050, it is predicted to reach 152 million people. It has evolved into a critical medical and social issue that must be addressed on a global scale since it has a significant detrimental impact on society and the economy [7].

AD is characterized by two pathological features: neurofibrillary tangles and extracellular amyloid aggregates [8,9,10,11]. The tau protein is abundant in neuronal axons and exerts toxic effects on cells through hyperphosphorylation and aggregation. Amyloid precursor protein (APP) is digested by β and γ secretase, yielding Aβ_40_ and Aβ_42_ [12,13,14]. Accumulation of Aβ_42_ damages synapses and causes cognitive decline [15]. Therefore, the development of inhibitors to block the aggregation of Aβ_42_ is one of the main approaches for the treatment of AD. However, the pace of drug development for Alzheimer’s disease is slow, and although drugs targeting Aβ can treat Alzheimer’s disease, they still face challenges [16]. Inhibitors of acetylcholinesterase have been found to be potentially helpful for the treatment of AD symptoms, but their use is very limited [17]. There are several types of Aβ peptide aggregation inhibitors, including organic molecules [18,19,20], peptides [21,22,23,24], plant extracts [25], antibodies [19], and nanoparticles [26,27]. However, they are usually associated with low binding affinities or brain permeability [28], and none of them are currently in clinical trials [17]. Small peptides have the advantage that they are more sensitive than larger molecules, and they are more likely to penetrate the blood–brain barrier. Additionally, the advantages of better biocompatibility, less toxic side effects, and small molecular weight, prompted us to investigate short peptides as potential therapeutic agents for preventing Aβ aggregation and for their therapeutic benefits in AD.

In this study, the polymerization mechanism of Aβ_42_ was analyzed by a molecular dynamics study. Molecular docking was used to screen dipeptides that can bind to Aβ_42_, using a dipeptide database comprised of the 20 L-α-amino acids, and molecular dynamics analysis was applied to examine the binding stability and interaction energy. Transmission electron microscopy, circular dichroism (CD), thioflavin T (ThT) fluorescence assay, MTT (3-[4,5-dimethylthiazol-2-yl]-2,5 diphenyl tetrazolium bromide) assay, and flow cytometry were conducted to investigate the effects of dipeptides on the aggregation and cell toxicity of Aβ_42_. It is hoped that the results of this study will open up new avenues for future Alzheimer’s disease studies.

## 2. Results

### 2.1. Molecular Dynamics Analysis of Aβ_42_ Conformation in a Dilute Sodium Chloride Solution

**Figure 1:**
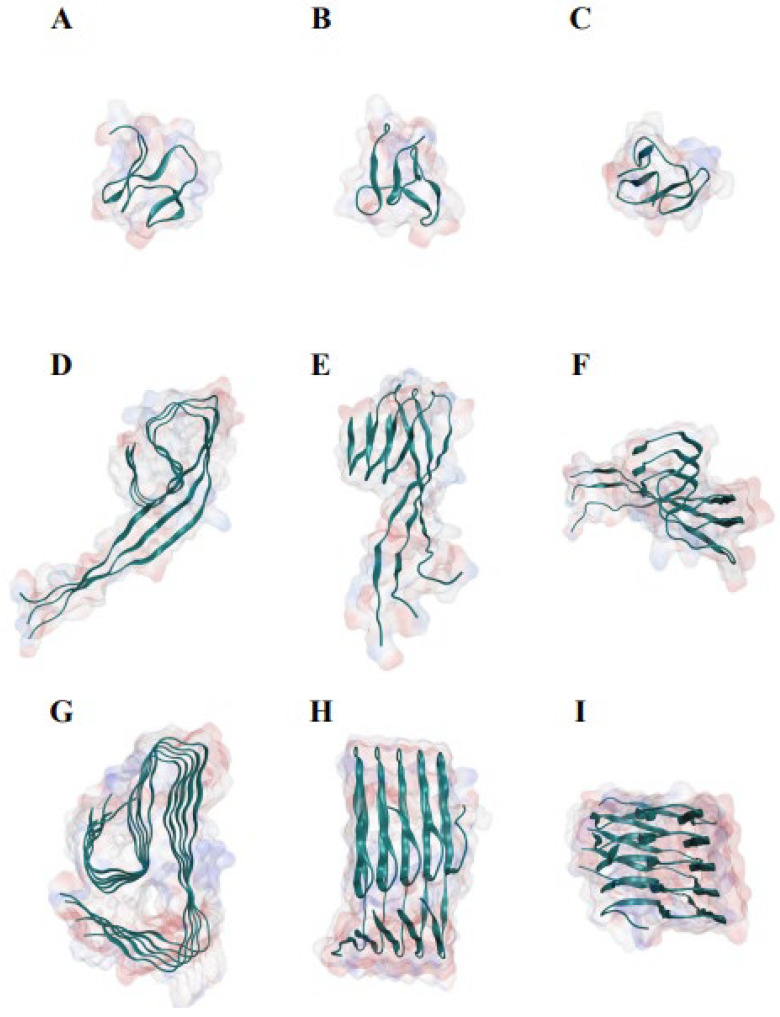
Molecular dynamics study of the structures of Aβ_42_ monomer, trimer, and pentamer in 0.1 M sodium chloride solution, demonstrating the association of Aβ_42_ polymerization with β-sheet conformation. (**A**) The front view of Aβ_42_ monomer. (**B**) The side view of Aβ_42_ monomer. (**C**) The top view of Aβ_42_ monomer. (**D**) The front view of Aβ_42_ trimer. (**E**) The side view of Aβ_42_ trimer. (**F**) The top view of Aβ_42_ trimer. (**G**) The front view of Aβ_42_ pentamer. (**H**) The side view of Aβ_42_ pentamer. (**I**) The top view of Aβ_42_ pentamer. The monomer, trimer, and pentamer of Aβ_42_ were modeled using the near atomic structure (PDB ID #5oqv) as starting conformations. Each analysis simulated 500-ns interaction and movements, with a data size of roughly 160 Gb.

In 0.1 M sodium chloride solution, Aβ_42_ monomer displayed a disordered conformation in MD analysis (Figure 1A–I; Supplementary Figure S1). The conformation of Aβ_42_ increasingly adopted the conformation observed by cryo-electron microscopy as the number of Aβ_42_ polymers increased [29] (Figure 1G–I). The Aβ_42_ monomer adopted a globular conformation made of β strands after a 1000 ns MD evaluation (Supplementary Figure S1). The hydrogen bonds around the N terminal of the Aβ_42_ trimer were broken, and the two sections were separated by the corner, twisted nearly 60 degrees (Figure 1D–F). The hydrogen bonds were substantially retained in the Aβ_42_ pentamer, and the angle between the two portions was greatly reduced (Figure 1G–I). Aβ_42_ adopts a conformation in which the backbone atoms are distributed almost in a flat plane in higher order polymers [29].

### 2.2. Molecular Dynamics Mechanisms Underlying the Aggregation of Aβ_42_

**Figure 2:**
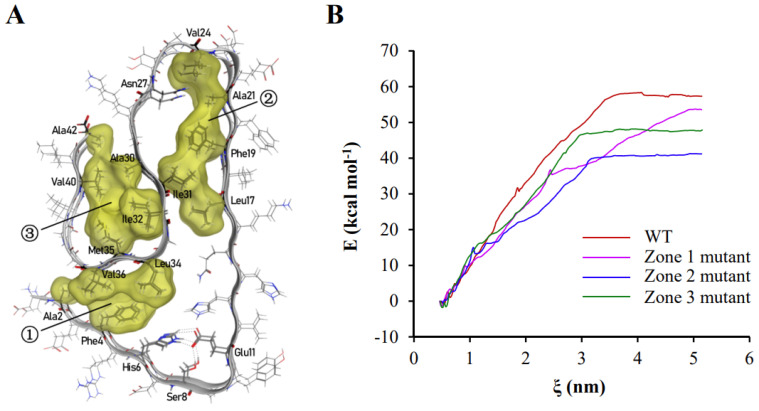
Hydrophobic interactions play a leading role in the polymerization of Aβ_42_. (**A**) In the polymerized Aβ_42_ conformation, there are three hydrophobic zones, which are indicated by the numbers in circles. Hydrophobic amino acid residues and those that create hydrogen bonding with side chains are labeled in the graph. (**B**) The effects of changing hydrophobic amino acids to glycine in the three hydrophobic zones on binding energy between two Aβ_42_ polypeptide chains. The binding energy of the wild-type Aβ_42_ dimer is represented by the red line; the binding energy of the first hydrophobic zone-mutated Aβ_42_ dimer is represented by the magenta line; the binding energy of the second hydrophobic zone-mutated Aβ_42_ dimer is represented by the blue line; and the binding energy of the third hydrophobic zone-mutated Aβ_42_ dimer is represented by the green line. The hydrophobic amino acid residues were labeled around each region. However, the residues that formed hydrogen bond were also labeled, including Asn27, His6, Ser8, and Glu11.

The majority of hydrophobic amino acid residues were trapped inside the polypeptide chain in aggregated Aβ_42_ polypeptides, resulting in the creation of three hydrophobic clusters (Figure 2A). Inside the polymers shown in Figure 1 and Figure 2A, the three hydrophobic clusters consist of (1) Ala2, Phe4, Leu34, Val36, (2) Leu17, Phe19, Ala21, Val24, Ile31, and (3) Ala30, Ile32, Met35, Val40, Ala42. As these hydrophobic clusters spread along the fibril axis inside of stacked subunits to be kept way from the solvents, they essentially help to keep the protein fibril structure stable. The contribution of the three hydrophobic zones to the binding energy between two polypeptide chains of Aβ_42_ were investigated by the umbrella sampling and the weighted histogram analysis method (WHAM) methods. These methods analyze the change in free energy along the reaction axis ζ when two Aβ_42_ polypeptide chains bound to each other are pulled apart by applying a harmonic force. The binding energy of two polypeptide chains of wild-type Aβ_42_ was 57.33 kcal·mol^−1^. The binding energy was reduced to 53.56 kcal·mol^−1^, 41.20 kcal·mol^−1^, and 47.88 kcal·mol^−1^ when the hydrophobic amino acid residues in the first, second, and third regions were changed to glycine, respectively (Figure 2B). These findings revealed that hydrophobic contact is the primary force driving Aβ_42_ aggregation through lowering free energy.

**Figure 3:**
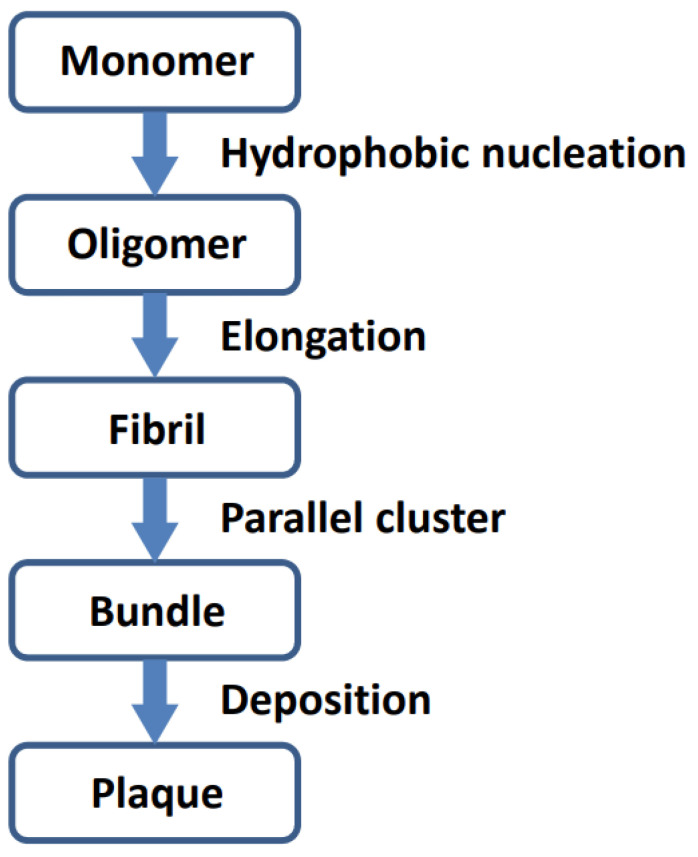
Schematic procedure of Aβ_42_ aggregation.

Analysis of the polymerized Aβ_42_ (5oqv) structure indicated the aggregation process of Aβ_42_ (Figure 3). The Aβ_42_ monomer first aggregates into oligomer seeds through hydrophobic interaction, and the Aβ_42_ polymer is further stabilized by hydrogen bonds between β strands. While oligomers are elongating, they form bundles through parallel clustering which may be driven by hydrophobic interaction and stabilized by electrostatic forces.

### 2.3. Analysis of the Interaction between Aβ_42_ and Dipeptides

**Figure 4:**
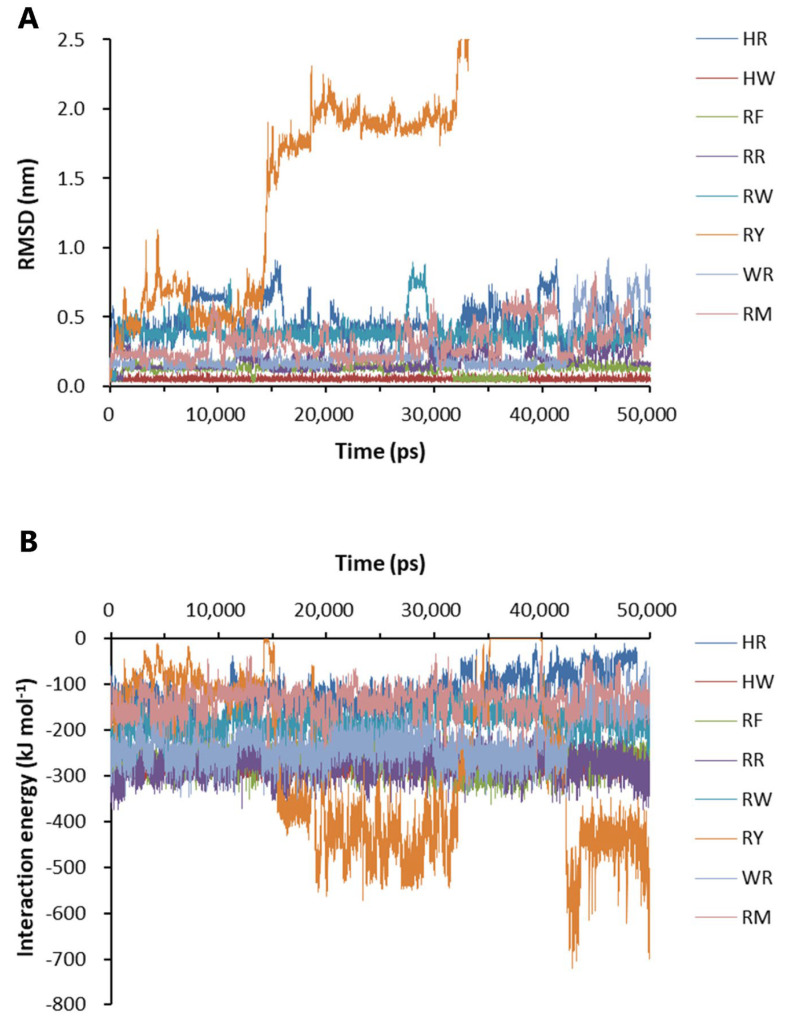
Binding stability and interaction energy of the dipeptides at different binding sites of Aβ_42_ pentamer. (**A**) The binding stability of dipeptides to Aβ_42_ pentamer. (**B**) The interaction energy between Aβ_42_ pentamer and the dipeptides. HR: Histidine–arginine; HW: Histidine–tryptophan; RF: Arginine–phenylalanine; RR: Arginine dipeptide; RW: Arginine–tryptophan; RY: Arginine–tyrosine; WR: Tryptophan–arginine; RM: Arginine–methionine. RMSD: The root-mean-square deviation of the positions of the heavy elements of a dipeptide at different binding sites over time. The interaction energy is the algebraic sum of Lennard-Jones and Coulombic potential energy.

Eight dipeptides were screened out by molecular docking. The docking position and conformation of the eight dipeptides are shown in Supplementary Figure S2. The change in the free energy of binding of the eight dipeptides to Aβ_42_ is shown in Table 1, which was calculated by the generalized Born volume integral/weighted surface area (GBVI/WSA) method. Based on the molecular docking results, the binding stability and interaction energy between the dipeptides and Aβ_42_ were analyzed. The binding of histidine–tryptophan dipeptide (HW) to Aβ_42_ was the most stable at the binding sites shown in Supplementary Figure S2. The root mean square of deviation (RMSD) value of the positions of the heavy elements of seven dipeptides bound to Aβ_42_ was maintained at a relatively low level during the 50-ns simulations, although there was fluctuation in the course (Figure 4A). The RMSD of RY reached the maximum value of 9.767 nm at 36.79 ns, indicating that RY was not able to associate for a longer time at the binding site (Figure 4A). The analysis of the interaction energy showed that RY was pulled away from the front docking site to another site with higher potential energy (Figure 4B). 

### 2.4. Effects of the Dipeptides on Aggregation of Aβ_42_

**Figure 5:**
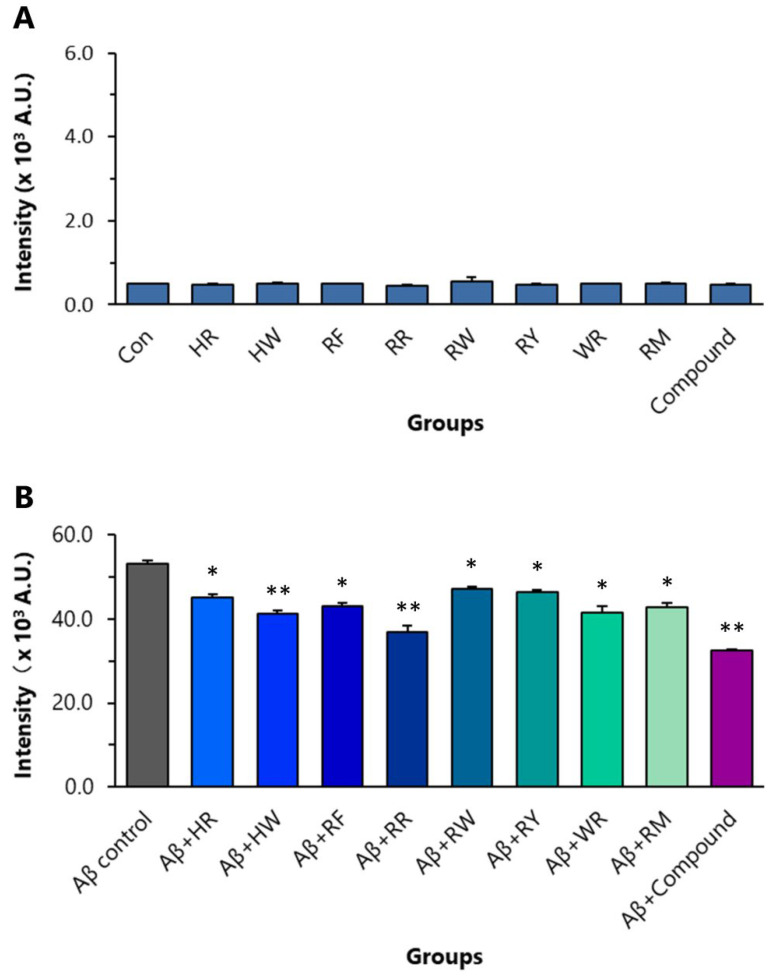
Effects of the eight dipeptides on aggregation of Aβ_42_. (**A**) Control experiment to show that dipeptides themselves have no effect on ThT fluorescent intensity. (**B**) The effects of the dipeptides on Aβ_42_ aggregation detected by ThT fluorometry. * *p* < 0.05 and ** *p* < 0.01 versus Aβ control group, analyzed by one-way ANOVA followed by the Tukey’s post-hoc test for multiple comparison, n = 5. HR: Histidine-arginine; HW: Histidine–tryptophan; RF: Arginine–phenylalanine; RR: Arginine dipeptide; RW: Arginine–tryptophan; RY: Arginine–tyrosine; WR: Tryptophan–arginine; RM: Arginine–methionine.

ThT binds to the extended structure of the β-sheet in amyloid fibrils with high specificity. In Aβ_42_ aggregation analysis, using ThT as an indication, all of the eight dipeptides (final conc. 8 µM) significantly inhibited the aggregation of Aβ_42_. In the control experiment, RR itself did not affect ThT fluorescent intensity (Figure 5A). RR at a final concentration of 8 µM inhibited Aβ_42_ (final conc. 10 µM) aggregation the most amongst the dipeptides (Figure 5B). The inhibitory effect of RR on the aggregation was stronger than that of HW (final conc. 8 µM). The compound composed of the eight dipeptides, each at a final concentration of 1 µM, generated stronger inhibitory effects compared with RR (Figure 5B).

### 2.5. Detailed Analysis of the Interaction between Aβ_42_ and Arginine Dipeptide

**Figure 6:**
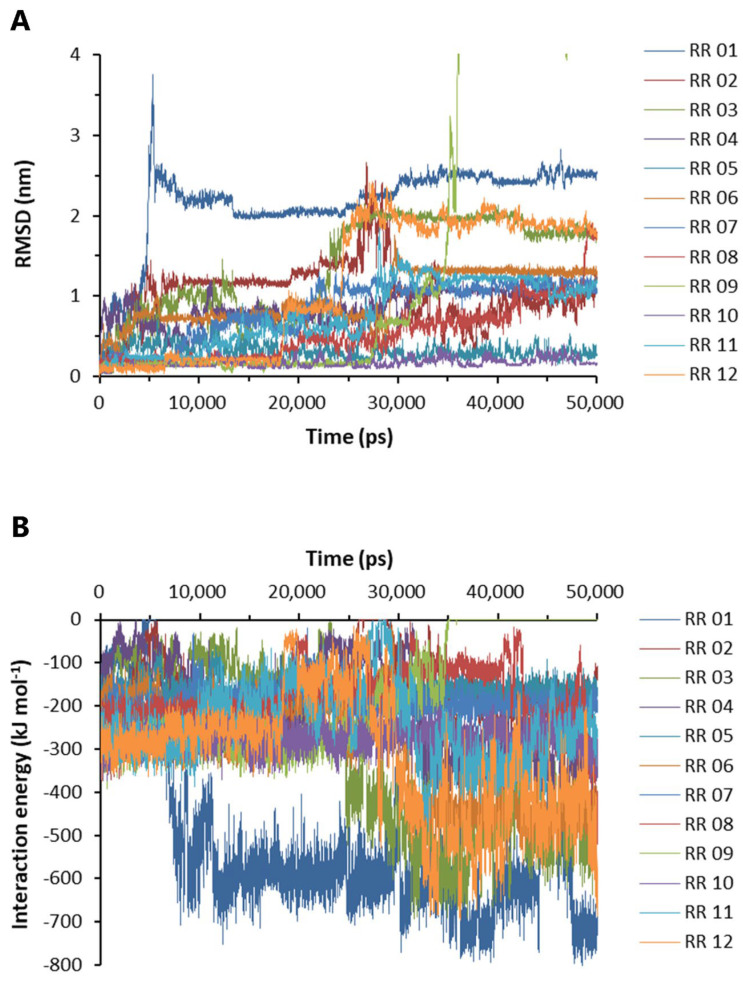
Binding stability and interaction energy of arginine dipeptide at different binding sites of Aβ_42_. (**A**) The root-mean-square deviation (RMSD) of the heavy elements of arginine dipeptide (RR) at different binding sites over time. (**B**) The interaction energy between Aβ_42_ pentamer and arginine dipeptide. The labels of RR 01-12 correspond to A to L in Supplementary Figure S3, respectively. The interaction energy is the algebraic sum of Lennard-Jones and Coulombic potential energy.

Based on the above experimental results, RR was selected for further molecular dynamics and pharmacological studies. The labeling of A to L in Supplementary Figure S3 corresponds to RR 01 to RR 12 in Figure 6, respectively. Nine docking configurations of RR to the Aβ_42_ pentamer (PDB #5oqv) are shown in Supplementary Figure S3, the structure of which was determined by NMR and cryo-electron microscopy [29]. Based on the docking results, the RR complexes with Aβ_42_ were subjected to molecular dynamics analysis. The root-mean-square deviation was evaluated by calculating the position of heavy elements in the RR structure to those in the starting structure. The RMSD value increases when the final structure deviates further from the initial structure; hence, the RMSD value is used to assess the binding stability of RR [30]. The RMSD values of RR from 12 binding conformations are shown in Figure 6A. The association at RR 10 and RR 05 sites was most stable (Figure 6A). RR 01 reached the highest RMSD value of 3.789 at 5.325 ns (Figure 6A). RR 09 reached the maximum RMSD value of 10.455 nm at 39.595 ns. The interaction energy between Aβ_42_ and RR was then examined, which is the algebraic sum of the Lennard-Jones and Coulombic potentials, with more negative IE values indicating a stronger pulling force between Aβ_42_ and RR (Figure 6B). The increase in IE of RR 01 and RR 09 over time suggested that they had moved to more stable locations (Figure 6B); however, when those sites were examined, they were not found to be on the front or back face of Aβ_42_.

**Figure 7:**
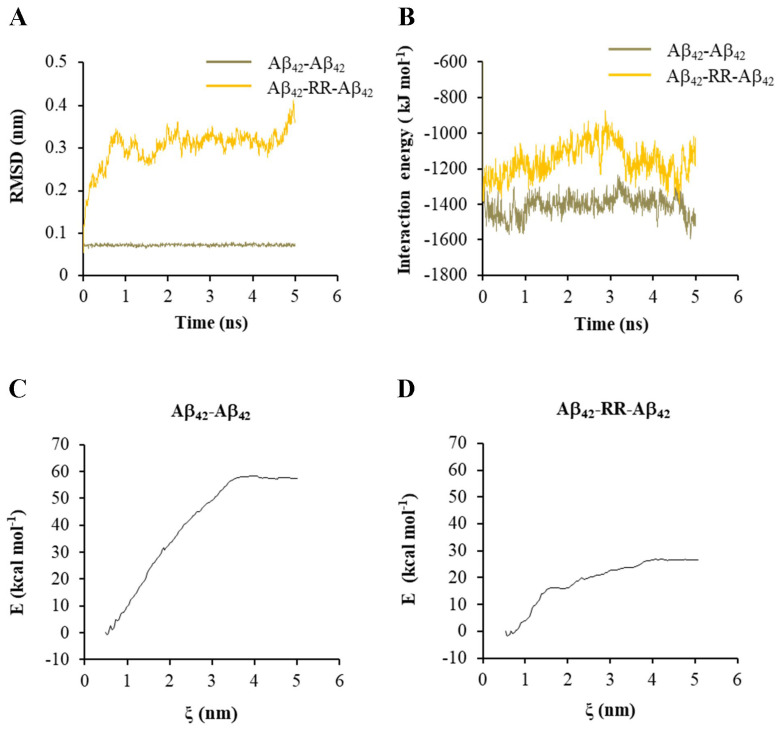
Effects of arginine dipeptide on the interaction between Aβ_42_ double strands. (**A**) The root-mean-square deviation (RMSD) of one of the Aβ_42_ double strands in absence or presence of arginine dipeptide (RR). (**B**) The interaction energy between Aβ_42_ double strands in absence or presence of RR. (**C**) The change in the free energy in the system when pulling the Aβ_42_ double strands apart along reaction axis ζ in absence of RR. (**D**) The change in the free energy in the system when pulling the Aβ_42_ double strands apart along reaction axis ζ in presence of RR.

With one of the Aβ_42_ strands being set to move freely, the RMSD of the free strand and IE between the two Aβ_42_ strands were analyzed. Without RR, the RMSD values of the free strands were low and varied at 0.07 nm, as shown in Figure 7A, indicating that the structure of the Aβ_42_ dimer was very stable. The RMSD values increased dramatically over time when a RR molecule was present, indicating that RR reduced the stability of the Aβ_42_ dimer structure (Figure 7A). The change in IE between Aβ_42_ strands in the presence or absence of a RR molecule is shown in Figure 7B. The absolute value of IE reduced when a RR molecule was present in between, indicating a decrease in the stability of the Aβ_42_ polymer. Meanwhile, umbrella sampling and WHAM analysis were conducted to examine the change in free energy in the system when the two strands of Aβ_42_ were pulled apart. When a RR molecule was present in the complex, the change in free energy of the system was greatly reduced, as shown in Figure 7C,D.

### 2.6. Effects of RR on Aβ_42_ Aggregation

**Figure 8:**
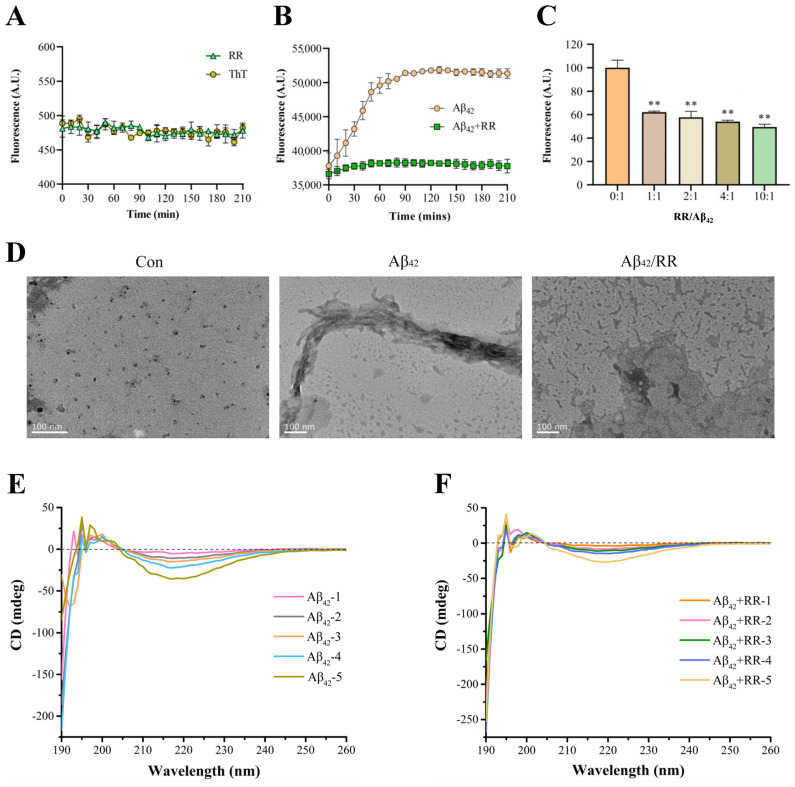
Effects of arginine dipeptide on aggregation of Aβ_42_. (**A**) Control experiment of the effects of arginine dipeptide on Thioflavin T (ThT) fluorescence intensity. At each data point, the sample size, n, is equal to 4. (**B**) Time-dependent effects of RR on Aβ_42_ aggregation detected by ThT fluorometry. Fluorescence intensity was measured at the wave length of 485 nm. *p* < 0.01, analyzed by one-way ANOVA, n = 4. (**C**) Dose-dependent effects of RR on Aβ_42_ aggregation detected by ThT fluorometry. Data are expressed as mean ± SE. ** *p* < 0.01 versus RR non-treated group, analyzed by one-way ANOVA followed by the Tukey’s post-hoc test for multiple comparison, n = 4. (**D**) Effects of arginine dipeptide (RR) on formation of Aβ_42_ fibrils detected by transmission electron microscopy. (**E**) Far-UV circular dichroism (CD) spectrometry of Aβ_42_ in absence of RR. Aβ_42_-1 to Aβ_42_-5 represents different concentration of Aβ_42_, which correspond to 12.5, 25, 50, 100, and 200 μM. (**F**) Far-UV circular dichroism (CD) spectrometry of Aβ_42_ in presence of RR. RR-1 to RR-5 represents different concentration of RR, which correspond to 31.25, 62.5, 125, 250, and 500 μM, respectively.

RR itself did not affect the ThT fluorescent intensity over time (Figure 8A). The fluorescence intensity of Aβ_42_ increased with time in the absence of RR, demonstrating the formation of amyloid fibril during incubation (Figure 8B). When RR was present in the solution, however, the fluorescence intensity of the Aβ_42_ solution remained lower, indicating that amyloid Aβ_42_ aggregation was reduced. This was also visible in the protection rate: at the 210-min mark, RR provided 26% protection. The fluorescence intensity of the samples was then measured using different doses of RR combined with Aβ_42_ (Figure 8C). The fluorescence intensity of Aβ_42_ reduced as the concentration of RR increased, and RR’s suppression of Aβ_42_ fluorescence intensity was dose-dependent. Therefore, RR is able to lower the quantity of amyloidogenic fibrils, effectively preventing amyloid aggregation formation.

A transmission electron microscope was used to investigate Aβ_42_ aggregation. Figure 8D displays TEM pictures of Aβ_42_ with and without RR. At 0 h, Aβ_42_ was mostly an amorphous structure with a significant number of distributed rows of granular protein molecules, and no evident aggregation or fibrous structures were seen. After 12 h of incubation without RR, TEM images revealed a high number of entangled fibrous aggregates, with protein molecules organized in needle-like aggregates and interlaced. Instead of huge tracts of fibrils, the aggregated form of Aβ_42_ protein revealed low-molecular aggregates with no fixed morphology in the Aβ_42_ samples co-incubated with RR, and its structure was drastically modified. This suggests that RR may have tampered with the aggregation pathway, preventing the formation of Aβ_42_ fibrils. These results indicate that RR significantly reduces the aggregated structure of Aβ_42_, and can effectively prevent the formation of fibrils.

Circular dichroism spectroscopy is a technique for evaluating protein secondary structures [31]. The circular dichroism spectra of Aβ_42_ protein with and without RR are shown in Figure 8E,F. The circular dichroism spectrum of Aβ_42_ shows a characteristic minimum at 217 nm, which is the β-sheet structure’s characteristic curve (Figure 8E). In the presence of RR, the intensity at 217 nm was decreased, indicating that the β-strand content was reduced and the random coil was increased (Figure 8F). There was a 62.8% decrease in β-sheet conformation and a 39.6% increase in random coiling of Aβ_42_ in the presence of RR (Table 2).

### 2.7. Effects of RR on Cell Toxicity of Secreted Aβ_42_

**Figure 9:**
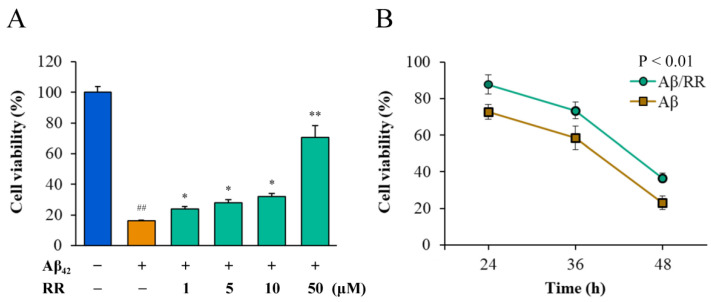
Effects of arginine dipeptide on cell toxicity induced by Aβ_42_ secreted from SH-SY5Y cells. (**A**) Effects of arginine dipeptide (RR) on cell toxicity induced by 48-h treatment with Aβ_42_ tested by MTT method. (**B**) Time-dependent effects of RR on cell toxicity of Aβ_42_. ^##^ *p* < 0.01 versus Aβ_42_ and RR non-treated group; * *p* < 0.05, ** *p* < 0.01 versus RR non-treated group, analyzed by one-way ANOVA followed by the Tukey’s post-hoc test for multiple comparison, n = 6.

MTT and CCK8 were used to detect differences in the viability of Aβ_42_-secreting SH-SY5Y cells in the absence and presence of RR (Figure 9). Normal cells that were not transfected with Aβ_42_-expressing plasmid were used as the blank control group. Without the addition of RR, most of the cells died 52 h after transfection with the plasmid. RR dose-dependently improved the viability of the cells secreting Aβ_42_ in doses ranging from 1 to 50 μM; at the highest concentration, the cell viability was more than 70% of that of the cells secreting Aβ_42_, indicating that RR can effectively protect cells and reduce the toxic effects related to Aβ_42_ (Figure 9A). The CCK-8 experiment showed that RR ameliorated the cell toxicity of Aβ_42_ over time (Figure 9B).

### 2.8. Effects of RR on the Secreted-Aβ_42_-Induced Increase in Reactive Oxygen Species

**Figure 10:**
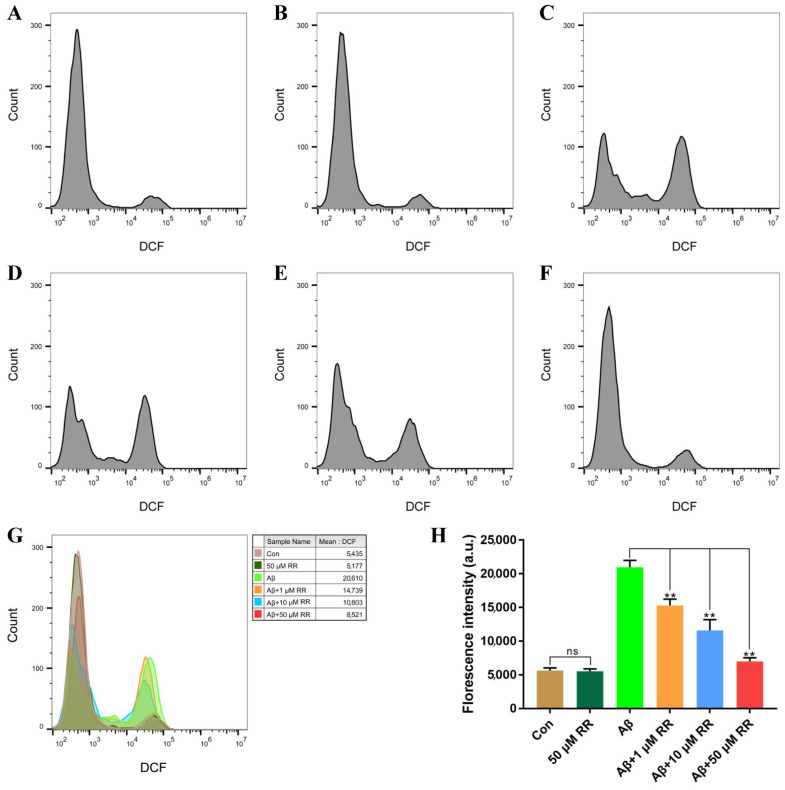
Flow cytometry analysis of the effects of arginine dipeptide on reactive oxygen species (ROS) production. (**A**) ROS production in Aβ_42_-nonsecreting SH-SY5Y cells treated with DMSO (Con). (**B**) ROS production in Aβ_42_-nonsecreting SH-SY5Y cells treated with arginine dipeptide (RR) at 50 μM. (**C**) ROS production in SH-SY5Y cells secreting Aβ_42_. (**D**–**F**) ROS production in Aβ_42_-secreting SH-SY5Y cells treated with RR at 1, 10, and 50 μM, respectively. (**G**) Overlay of the flow cytometric figures, (**A**–**F**). (**H**) Quantification of ROS production. All experiments were repeated at least three times. Results are expressed as mean ± SEM, ns: not significant, ** *p* < 0.01, analyzed by one-way ANOVA, followed by the Tukey’s post-hoc test for multiple comparison.

The production of ROS in SH-SY5Y cells was evaluated after 48 h of treatment. Compared to Aβ_42_-nonsecreting SH-SY5Y cells, Aβ_42_-secreted from SH-SY5Y cells increased the ROS levels in the cells from 5630 ± 460 to 20,959.67 ± 1161.33. This represented a 3.72-fold increase (Figure 10). Treating the cells with RR at final concentrations of 1, 10, and 50 μM significantly ameliorated the increase in the ROS level induced by the secreted Aβ_42_, at 15,271.67 ± 1124.33, 11,583.33 ± 1855.67, and 6982.33 ± 600.67, respectively (Figure 10).

### 2.9. Effects of RR on Cell Apoptosis Induced by Secreted Aβ_42_

**Figure 11:**
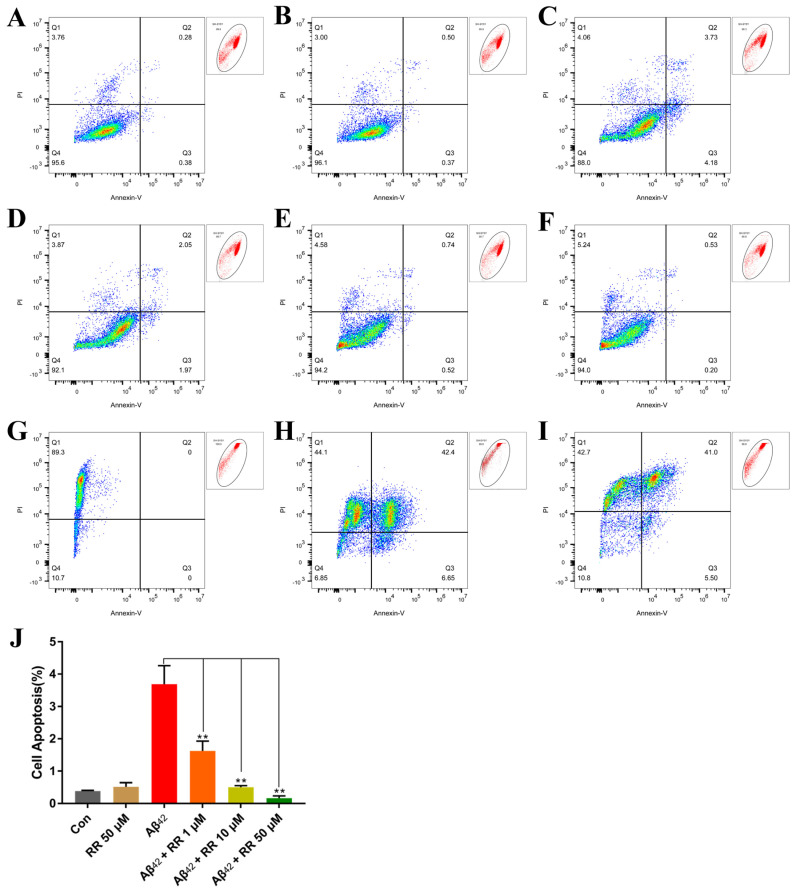
Flow cytometry analysis of the effects of arginine dipeptide on cell apoptosis. (**A**) Apoptosis in Aβ_42_-nonsecreting SH-SY5Y cells treated with control medium (Con). (**B**) Apoptosis in Aβ_42_-nonsecreting SH-SY5Y cells treated with arginine dipeptide (RR) at 50 μM. (**C**) Apoptosis in SH-SY5Y cells secreting Aβ_42_. (**D**–**F**) Apoptosis in Aβ_42_-secreting SH-SY5Y cells treated with RR at 1, 10 and 50 μM of Aβ_42_, respectively. (**G**–**I**) SH-SY5Y cells incubated with PI, Alexa Fluor 488 annexin V conjugate, and both of them, respectively. (**J**) Quantification of cell apoptosis with FlowJo^TM^. Results are expressed as mean ± SEM. ** *p* < 0.01, analyzed by one-way ANOVA, followed by the Tukey’s post-hoc test for multiple comparison, n = 13.

Compared to Aβ_42_-nonsecreting cells, Aβ_42_ secreted from SH-SY5Y cells greatly increased apoptosis in the cells from 0.387 ± 0.012 (mean ± SEM) percent to 3.687 ± 0.330 percent. This represented a 9.52-fold increase (Figure 11). Treating the cells with RR at final concentrations of 1, 10, and 50 μM significantly ameliorated the apoptosis induced by the secreted Aβ_42,_ and the percentage of apoptotic cells reached 1.623 ± 0.175, 0.5 ± 0.031, and 0.163 ± 0.042 percent, respectively (Figure 11). 

## 3. Discussion

Protein aggregation and misfolding are complicated processes [32,33]. For example, the imbalance between the production, accumulation, and clearance of Aβ_42_ contributes to the pathological changes in AD, and understanding how amyloid structures change is a challenge in developing therapies for AD [34,35]. Molecular dynamics is one of the tools that provide the necessary spatial and temporal resolution to study the interactions between amyloid molecules [36]. In this study, molecular dynamics analysis was performed on Aβ_42_ in various polymerization phases, and three hydrophobic clusters were discovered in the Aβ_42_ structure by altering the protein structure. The binding energy variations of the two polypeptide chains of Aβ_42_ in the umbrella sample were compared after all amino acids in each hydrophobic cluster were changed to glycine. The binding energy of the mutant Aβ_42_ dimer was lower than that of the wild-type Aβ_42_ dimer, and its structural stability was altered, showing that the hydrophobic clusters play a key role in Aβ_42_ protein polymerization.

Structure-based drug discovery is possible for amyloid diseases, just as it is for other medical diseases [37]. Using a dipeptide library, molecular docking of Aβ_42_ was performed, and arginine dipeptide was screened. The interactions between the Aβ_42_ protein receptor and RR were analyzed. RR could generate hydrogen-bonding interactions with some amino acid residues of Aβ_42_ at some sites with different conformations. In some specific conformations, the RMSD value was the smallest and remained stable throughout, and RR could bind most tightly to the Aβ_42_, forming the most stable complex structure. Moreover, the IE values of the complex stayed at the most negative level compared to other conformations.

In the absence of RR, the Aβ_42_ double-strand structure was very stable in water, with a very low and steady RMSD value. However, in the presence of RR, the RMSD increased dramatically. Furthermore, the strand structure altered from the initial state, indicating that the structure stability was degraded. At the same time, the absolute value of the interaction energy was decreased. In umbrella sampling, when RR was present in the system, the energy required to pull apart the Aβ_42_ double strands was reduced, indicating that the binding of the Aβ_42_ double strands was interfered with by RR.

Using approaches well established in amyloid formation studies, including ThT fluorescence experiments, transmission electron microscopy, and circular dichroism spectroscopy, we investigated the possibility that RR alters the kinetics of Aβ_42_ aggregation and the formation of fibrils from Aβ_42_ monomers in vitro. The fluorescence intensity of Aβ_42_ treated with RR was significantly lower than that of Aβ_42_ in the ThT experiment, and RR altered the kinetics of Aβ_42_ protein aggregation. Co-incubated Aβ_42_ with different concentrations of RR resulted in the fluorescence intensity of Aβ_42_ declining with the increase in RR concentration; furthermore, the inhibitory effect of RR was dose-dependent [38,39,40,41]. Then, we evaluated how RR altered the secondary structure of the Aβ_42_ protein. The β-sheet content of Aβ_42_ reduced by 62.8% in the presence of RR and the content of random coils increased by 39.6%, which appear mostly in a non-aggregated conformation of Aβ_42_ [42]. As demonstrated in Figure 1, the development of a β-sheet structure resulted from polymerization, and vice versa. [43,44]. Aβ_42_ co-cultured with RR exhibited morphological alterations, as well as a decrease in the quantity of amyloidogenic fibers, as indicated in the transmission electron micrograph. 

## 4. Material and Methods

Aβ_42_ was purchased from ChinaPeptides Co., Ltd. (Shanghai, China) and dissolved in a 1.0% NH_4_OH solution to a stocking concentration of 1 mM. Dipeptides were synthesized by Sangon Biotech Co., Ltd. (Shanghai, China) and dissolved in a phosphate buffer (10 mM, pH 7.4, PBS) to a stocking concentration of 1 mM. All stocking solutions were aliquoted and stored at –80 °C. All protein solutions were diluted in PBS in experiments. ThT and MTT were purchased from Sigma-Aldrich (St. Louis, MI, USA).

### 4.1. Molecular Dynamics Analysis of the Structure of Aβ_42_ Monomer, Trimer and Pentamer

Molecular dynamics studies were conducted using the Groningen Machine for Chemical Simulation (GROMACS, 2020.03) on the Ubuntu (18.06) Linux operating system, and was accelerated by NVIDIA Compute Unified Device Architecture (CUDA)-supported parallel computation. The Aβ_42_ monomer, trimer or pentamer was placed at the center of a dodecahedron box with a distance of 3.0 nm from the edge to the Aβ_42_. The box was filled with water molecules, and neutralized by randomly replacing water molecules with Na^+^ and Cl^−^ at final concentrations of 0.1 M. The starting conformations of the Aβ_42_ monomer, trimer, and pentamer were derived from the Aβ_42_ polymer structure (PDB #5oqv), which was determined by cryo-electron microscopy [29]. The Amber99SB force field, which is optimized for the ab-initio calculation of three-dimensional structure of proteins, and the TIP3P explicit water model were used throughout the MD study.

Energy minimization was carried out using the steepest descent algorithm, stopping when the maximal force was less than 1000 kJ/mol/nm. After energy minimization, the system was equilibrated under an isothermal–isochoric ensemble using the velocity-rescaling method to ensure a correct kinetic energy distribution by modifying the standard Berendsen thermostat in accordance with Equation (1):

(1) $$dK=\left(K_{0}-K\right)\frac{dt}{\tau_{T}}+2\sqrt{\frac{KK_{0}}{N_{f}}}\;\frac{dW}{\sqrt{\tau_{T}}}$$

where *K* is the kinetic energy, *N_f_* is the number of degrees of freedom, d*W* is a Wiener stochastic process, and *τ_T_* is close to the time constant *τ* of the temperature coupling, and is given by Equation (2):

(2) $$\tau =2C_{V}\tau_{T}/N_{df}k$$

The system was then equilibrated using the Parrinello–Rahman pressure coupling algorithm to give a true isothermal–isobaric ensemble. The volume of the unit cell (*V*) was given by Equation (3):

(3) $$\frac{{\mathrm{db}}^{2}}{dt^{2}}=VW^{-1}b^{\prime -1}\left(P-P_{ref}\right)$$

where P is the pressure, and *W* is the strength of pressure coupling, which can be calculated by Equation (4):

(4) $${\left(W^{-1}\right)}_{ij}=\frac{4\pi^{2}\beta_{ij}}{3\tau_{p}^{2}L}$$

where *τ_P_* is the pressure time constant, *β* is the isothermal compressibility which was 4.5 × 10^−5^/bar, and *L* is the largest box matrix element. 

After equilibration, the MD analysis was performed in 250,000,000 time steps, yielding a data size of approximately 160 Gb. In each time step, short range interaction including both van der Waals and electrostatic forces were derived from the Lennard-Jones (LJ) (5) and Coulombic (Coul) potentials (6), and the Verlet cutoff-scheme with a buffer size of 5 × 10^−3^ kJ/mol/ps was applied.

(5) $$F_{LJ\left(ij\right)}=-\left(12\frac{C_{ij}^{\left(12\right)}}{r_{ij}^{13}}-6\frac{C_{ij}^{\left(6\right)}}{r_{ij}^{7}}\right)\frac{r_{ij}}{r_{ij}}$$

(6) $$F_{Coul\left(ij\right)}=-f\frac{q_{i}q_{j}}{\varepsilon_{r}r_{ij}^{2}}\frac{r_{ij}}{r_{ij}}$$

where *F_ij_* is the force on atom *i* exerted by atom *j*, **r** is the position vector, *r* is the vector length, *q* is the elementary charge equals to 1.602176565 × 10^−19^ *C*, and *f* is the electric conversion factor, which equals 1/4π*ε*_0_ or 138.935458 kJ mol^−1^ nm e^−2^.

The smooth particle mesh Ewald (PME) summation was used to compute the long-range electrostatic interaction. Using cardinal B-spline interpolation, the charges were assigned to a grid, which was then transformed using the three-dimensional fast Fourier algorithm. The electrostatic force was back converted from the reciprocal force at position **r***_αi_*, *α* = 1, 2, 3, which is a differentiation of the reciprocal energy Equation (7):

(7) $${\tilde{E}}_{\mathrm{rec}}=\frac{1}{2}\;\overset{K_{1}-1}{\underset{m_{1}=0}{\sum }}\overset{K_{2}-1}{\underset{m_{2}=0}{\sum }}\overset{K_{3}-1}{\underset{m_{3}=0}{\sum }}Q\left(m_{1},m_{2},m_{3}\right)\cdot \left(\theta_{\mathrm{rec}}*Q\right)\left(m_{1},m_{2},m_{3}\right)$$

where *m* is the reciprocal lattice vector, *Q* is the array of vectors, and *θ*_rec_ is the pair potential.

### 4.2. Molecular Dynamics Analysis of Binding Energy

The computational system was the same as that stated above. The change in Gibbs free energy (∆G) that occurs as a ligand is pulled away along a reaction axis (ξ) was calculated using umbrella sampling and the WHAM [45]. The ∆G indicates the binding energy between the protein and the ligand in such an ensemble. The method was adapted from our previous publication [46]. The simulation cell with periodic boundaries was a rectangular box with the dimension of 6.0 × 7.0 × 14.0 nm, and the Aβ_42_ protein complex bound to the dipeptide was centered at 3.0, 3.5, 1.5 (x, y, z). The cell was filled with water molecules and neutralized with sodium chloride^−^ at final concentrations of 0.1 M. Reference atoms were set to the carbonyl carbon atom of the 29^th^ glycine, and the Cα of the first amino acid of a dipeptide. Before the pulling and umbrella sampling stages, the pressure equilibration shown above was employed. In the step of generating configurations, the two proteins were pulled away by applying a harmonic force at a constant velocity of 0.01 nm/ps over a course of 250,000 time steps, and 501 coordinate files were saved along the pulling. Then, twenty-three to twenty-five umbrella samplings, each of 10 ns, in overlapping 0.2-nm-spacing windows along the ξ axis, were made, yielding approximately 448 Gb of data. The ∆G was calculated using the WHAM module of GROMACS [47,48].

### 4.3. Molecular Docking

A ChemScript written in Python was executed to build chemical structure database consisting of all the dipeptides made from the 20 L-α-amino acids. The Aβ_42_ structure in an aggregated conformation was downloaded from the Protein Data Bank (PDB, #5oqv). Automated molecular docking was carried out using AutoDock. The ligand placement was carried out using the triangle matcher and evaluated by the Longdon dG scoring. The induced fit method was applied to refine the docked structures, and the GBVI/WSA dG method was used for ranking.

### 4.4. Ligand Interaction Analysis

The computational system stated above was adopted for the analysis, using the AMBER99SB force field and explicit TIP3P water model. A dodecahedron box was defined, and the Aβ_42_ complex with a dipeptide ligand was placed at the center of the box, which was then filled with water and neutralized with Na^+^ and Cl^−^ ions. The temperature was stabilized by NVT equilibration after the energy in the system was minimized, and pressure equilibration was conducted under an NPT ensemble. To assess the binding stability of a dipeptide to Aβ_42_, the RMSD of the position of the heavy atoms in a dipeptide was determined by Equation (8):

(8) $$RMSD_{t_{1},t_{2}}={\left[\frac{1}{M}\overset{N}{\underset{i=1}{\sum }}m_{i}||r_{i}\left(t_{1}\right)-r_{i}\left(t_{2}\right)|{|}^{2}\right]}^{\frac{1}{2}}$$

where **r***_i_* is the position vector of atom *i*, *t* represents time, and *M* is the summation of atom mass (*m*).

The interaction potential energy is the sum of the Lennard-Jones potential (9) and the Columbic energy (10) determined by the equations:

(9) $$E_{LJ\left(ij\right)}=\frac{C_{ij}^{\left(12\right)}}{r_{ij}^{12}}-\frac{C_{ij}^{\left(6\right)}}{r_{ij}^{6}}$$

(10) $$E_{Coul\left(ij\right)}=f\frac{q_{i}q_{j}}{\varepsilon_{r}r_{ij}}$$

where *r* is the length of position vector, q is the elementary charge, which equals 1.602176565 × 10^−19^ *C*, and *f* is the electric conversion factor which equals 1/4πε_0_ or 138.935458 kJ mol^−1^ nm e^−2^. The Lennard-Jones and Columbic potentials between all Aβ_42_ and dipeptides atoms were summed to obtain the overall interaction energy between Aβ_42_ and dipeptides.

### 4.5. Transmission Electron Microscopy

Transmission electron microscopy was adopted to observe the morphology of Aβ_42_ fibers. Briefly, 100 µM of Aβ_42_ solutions was incubated with 500 µM of arginine dipeptide (RR) at 37 °C for 12 h. After incubation, 5 µL of the incubated solution was loaded onto Formvar/carbon-coated grids, negatively stained with 1% phosphotungstic acid, and air dried at room temperature. The stained samples were observed using a transmission electron microscope (JEOL, Tokyo, Japan).

### 4.6. Circular Dichroism Spectroscopy

Circular dichroism (CD) spectroscopy was applied to analyze the changes in the secondary structure of Aβ_42_ in the presence or absence of RR. In the experiment, Aβ_42_ was incubated with RR at different concentration at 37 °C for 24 h. Far-UV CD spectra were recorded from 190 to 260 nm, and the scan rate was 100 nm/min. Each sample was scanned three times to obtain an average CD spectrum. The spectral data were analyzed by using the DichroWeb server to predict the percentage of α-helix, β-fold, and random coils in Aβ_42_ [49,50].

### 4.7. Thioflavin T Fluorometric Assay 

Thioflavin T (ThT) fluorometric assay was used to detect real-time aggregation of Aβ_42_ in the absence or presence of RR. Thioflavin was dissolved to obtain a stocking solution of 4 mM in 10 mM of PBS. The final concentration of Aβ_42_, RR and ThT was 10, 200, and 16 µM. Fluorescence intensity was measured at 485 nm on a Synergy H1 multimode microplate reader (Agilent, Santa Clara, CA, USA) with an excitation wavelength of 450 nm every 5 min.

To analyze the dose-dependent effects of RR on Aβ_42_ aggregation, the RR stock solution was added at a final concentration of 10, 20, 40, and 100 μM; then, Aβ_42_ was added at a final concentration of 10 μM. The mixture was incubated at 37 °C for 24 h; then, ThT at a final concentration of 16 µM was added, and the fluorescence intensity was measured at 485 nm on the microplate reader. 

### 4.8. MTT Assay

The SH-SY5Y cell line was transfected with a pcDNA3.1 plasmid expressing secreted Aβ_42_ (N-terminal plus secretory signal peptide) for 4 h and divided into the wells of 96-well plates according to the number of cells 5 × 10^3^. The experimental groups were added with a final concentration of 1, 5, 10, and 50 μM of RR. The cells not transfected with the Aβ_42_ expression plasmid were used as the blank control group, and cells without RR were used as the Aβ control group. After 48 h incubation at 37 °C in a 5% CO_2_ incubator, the medium was aspirated, 90 μL of serum-free medium and 10 μL of MTT solution (5 mg/mL) were added to each well. After 4 h, the wells were aspirated, 100 μL of DMSO was added, and the wells were shaken for 10 min at 37 °C. The absorbance values of each well were measured at 570 nm in a Synergy H1 microplate reader, and the mean value of the blank control was used as the 100% reference value to calculate the cell viability of each well.

### 4.9. CCK-8 Test

Cells transfected with the pcDNA3.1 plasmid expressing secreted Aβ_42_ (N-terminal plus secretory signal peptide) were seeded in 96-well plates (2 × 10^4^/well), and incubated with or without RR at a final concentration of 10 μM at 37 °C for 0, 24, 36, and 48 h. Then, cells were further incubated with 10 μL of CCK-8 solution provided in the kits at 37 °C for an additional 3 h. Cell viability was tested by measuring the absorbance of UV at a wave length of 450 nm.

### 4.10. Flow Cytometric Analysis on Apoptosis of SH-SY5Y Cells Secreting Aβ_42_

The SH-SY5Y cells overexpressing Aβ_42_ were cultured with RR at 1, 10, and 50 μM for 48 h. Then, the cells were collected and washed with PBS. After washing, the cells were stained using PI and the Annexin-V kit (UElandy Inc., Suzhou, China), and cell apoptosis was measured by flow cytometry (CytoFLEX LX, Beckman Coulter Life Sciences, Indianapolis, IN, USA). The data were analyzed using FlowJo^TM^ (BD Biosciences, San Jose, CA, USA). Statistical results of three independent experiments were expressed as the mean ± SEM. 

### 4.11. Statistical Analysis

All experimental data were statistically analyzed using Prism 8 (GraphPad, Boston, MA, USA), and/or plotted using Origin 2016 (OriginLab, Northampton, MA, USA). A *p*-value of less than 0.05 was considered to be statistically significant. 

## 5. Conclusions

The hydrophobic force is the leading factor in polymerization and folding of Aβ_42_, which are two sides of the same process to reduce free energy in a water solution. According to the results of molecular docking and dynamics analyses, RR forms hydrogen bonds with the backbone atoms of Aβ_42_ oligomers to inhibit it elongating into fibrils. According to the results of this study, RR may be beneficial in preventing the progress of AD.

## 6. Patents

A patent application with the application number 202210872429.4 is pending.

## Figures and Tables

**Table 1:** The free energy of binding of the dipeptides from the poses shown in Supplementary Figure S2.

Dipeptides	ΔG * kcal mol^−1^
HR	−26.2075
HW	−42.5297
RF	−35.7649
RR	−38.5242
RW	−35.8093
RY	−36.7211
WR	−30.7953
RM	−33.6804

* The free energy was calculated using the GBVI/WSA method.

**Table 2:** Predicted secondary structures based on the CD test.

	Secondary Structures (%)			
	Helix	Strand	Turn	Disordered
Aβ_42_	3.0	29.8	23.4	42.7
Aβ_42_ + RR	0.8	11.1	28.0	59.6

## Data Availability

All data and materials related to the study are available upon request.

## Supplementary Materials

The following supporting information can be downloaded at: https://www.mdpi.com/article/10.3390/ijms24087673/s1.

## Abbreviation

Aβ_42_	Amyloid β peptide (1–42)
AD	Alzheimer’s disease
CD	Circular dichroism
CUDA	Compute unified device architecture
ΔG	Change in Gibbs free energy
GROMACS	Groningen Machine for Chemical Simulation
IE	Interaction energy
MD	Molecular dynamics
MTT	3-(4,5-dimethylthiazole-2-yl)-2,5-diphenyltetrazolium bromide
PDB	Protein data bank
RMSD	Root-mean-square deviation
RR	Arginine dipeptide
TEM	Transmission electron microscopy
ThT	Thioflavin T
