# Supplementary material for: Molecular Integrative Analysis of the Inhibitory Effects of Dipeptides on Amyloid β Peptide 1–42 Polymerization
Source: Int J Mol Sci. 2023 Apr 21;24(8):7673. doi: 10.3390/ijms24087673 (PMC10141046; doi:10.3390/ijms24087673)
Supplement: Supplementary file 1 [file ijms-24-07673-s001.zip › ijms-2292052-supplementary.pdf]

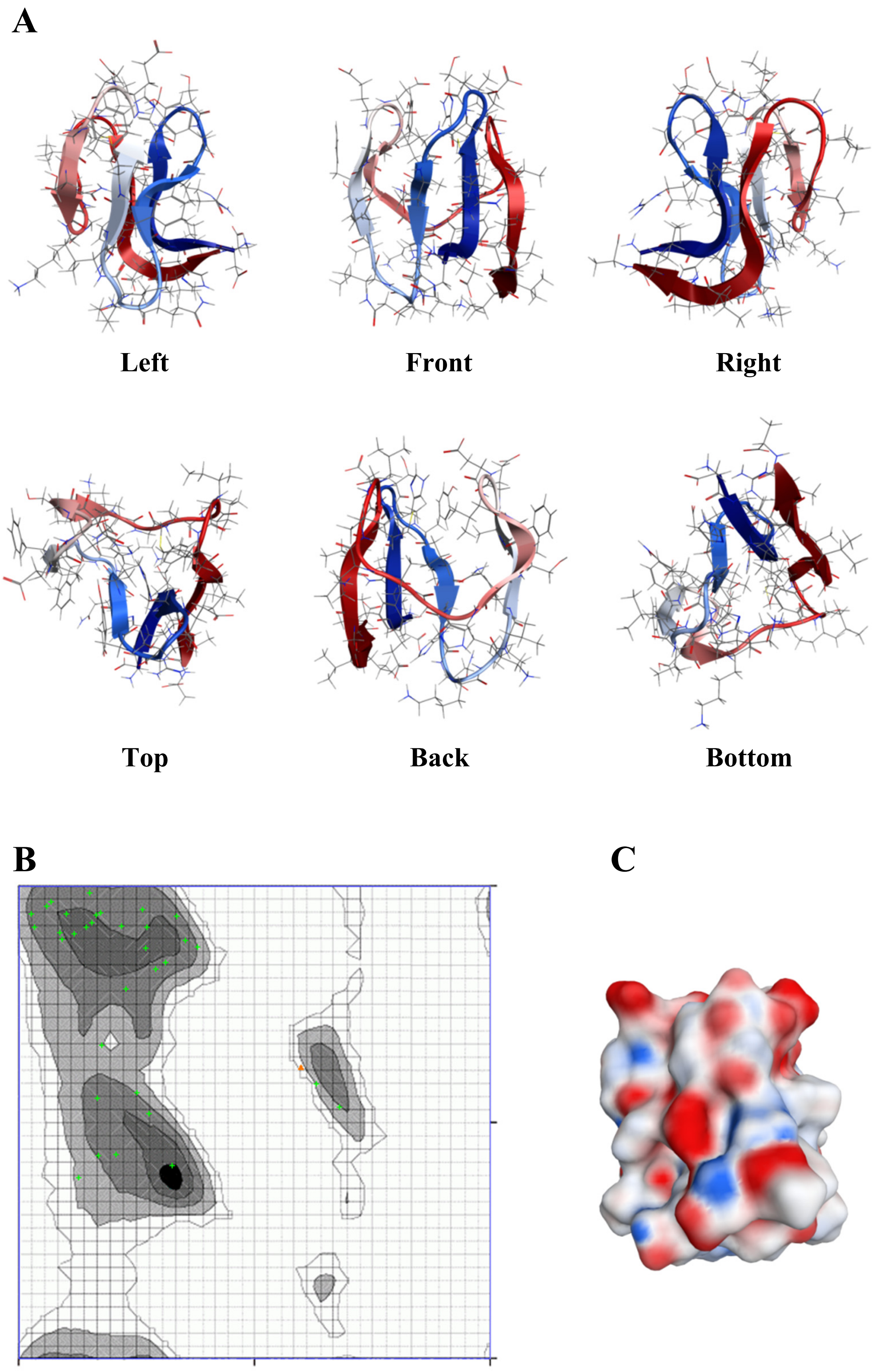

Supplementary Figure S1. Molecular dynamics analysis of the **c**onformation of Aβ_{42} monomer in dilute sodium chloride solution. **A.** The conformation of Aβ_{42} in 0.1 M sodium chloride solution after 1000 ns simulation by GROMACS using the AMBER99SB force field and TIP3P explicit water model. **B.** Ramachandran plot of dihedral angles of the Aβ_{42} monomer. **C.** Electrostatic distribution on the surface of Aβ_{42} monomer.

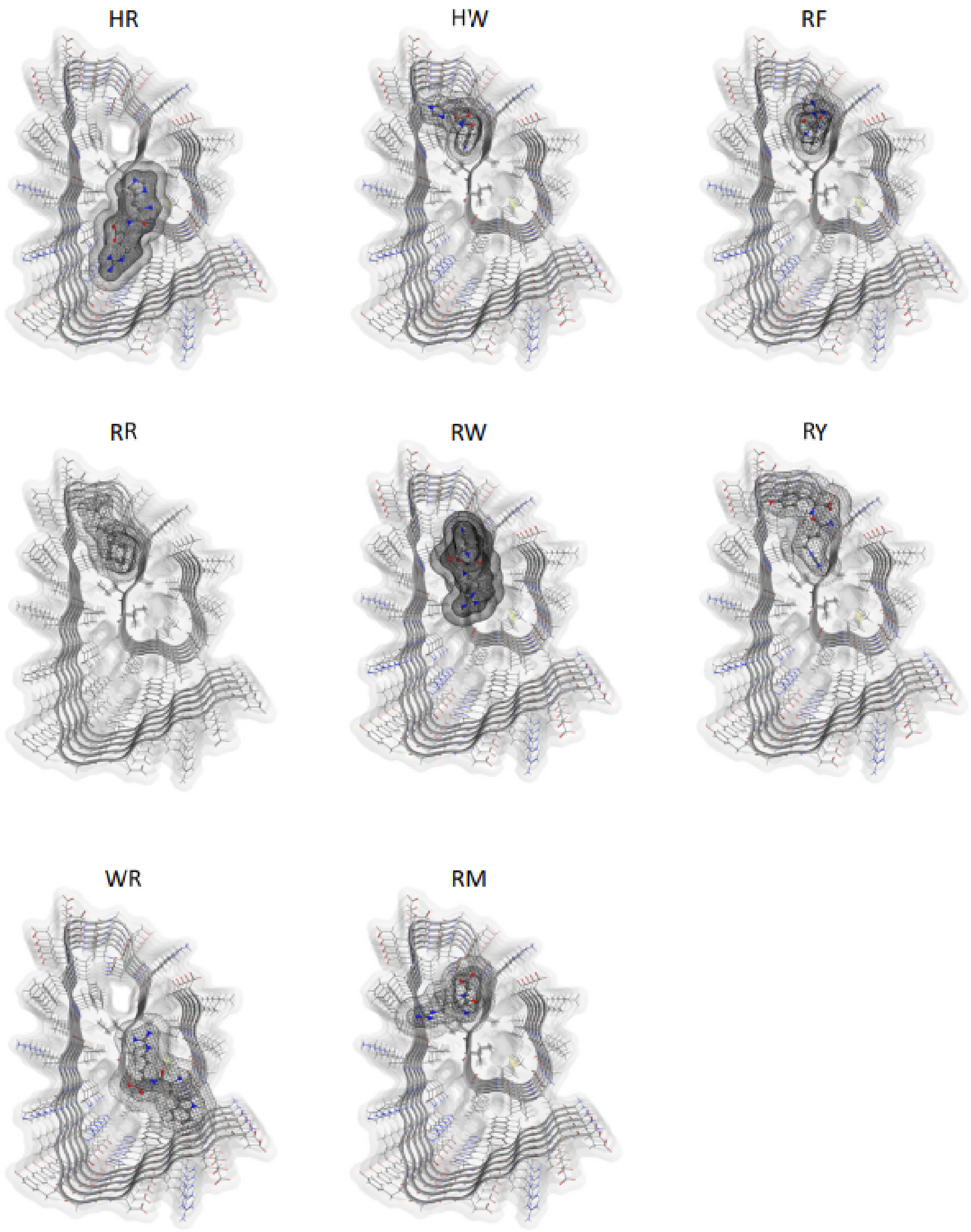

Supplementary Figure S2. Docking position of eight dipeptides on Aβ_{42} pentamer. The eight dipeptides bound to Aβ_{42} was screened out by molecular docking. HR: Histidine-arginine; HW: Histidine-tryptophan; RF: Arginine-phenylalanine; RR: Arginine dipeptide; RW: Arginine-tryptophan; RY: Arginine-tyrosine; WR: Tryptophan-Arginine; RM: Arginine-methionine. The molecular surface of dipeptides and the boundary of van der Waal's force were indicated by dark and light grey mesh diagrams.

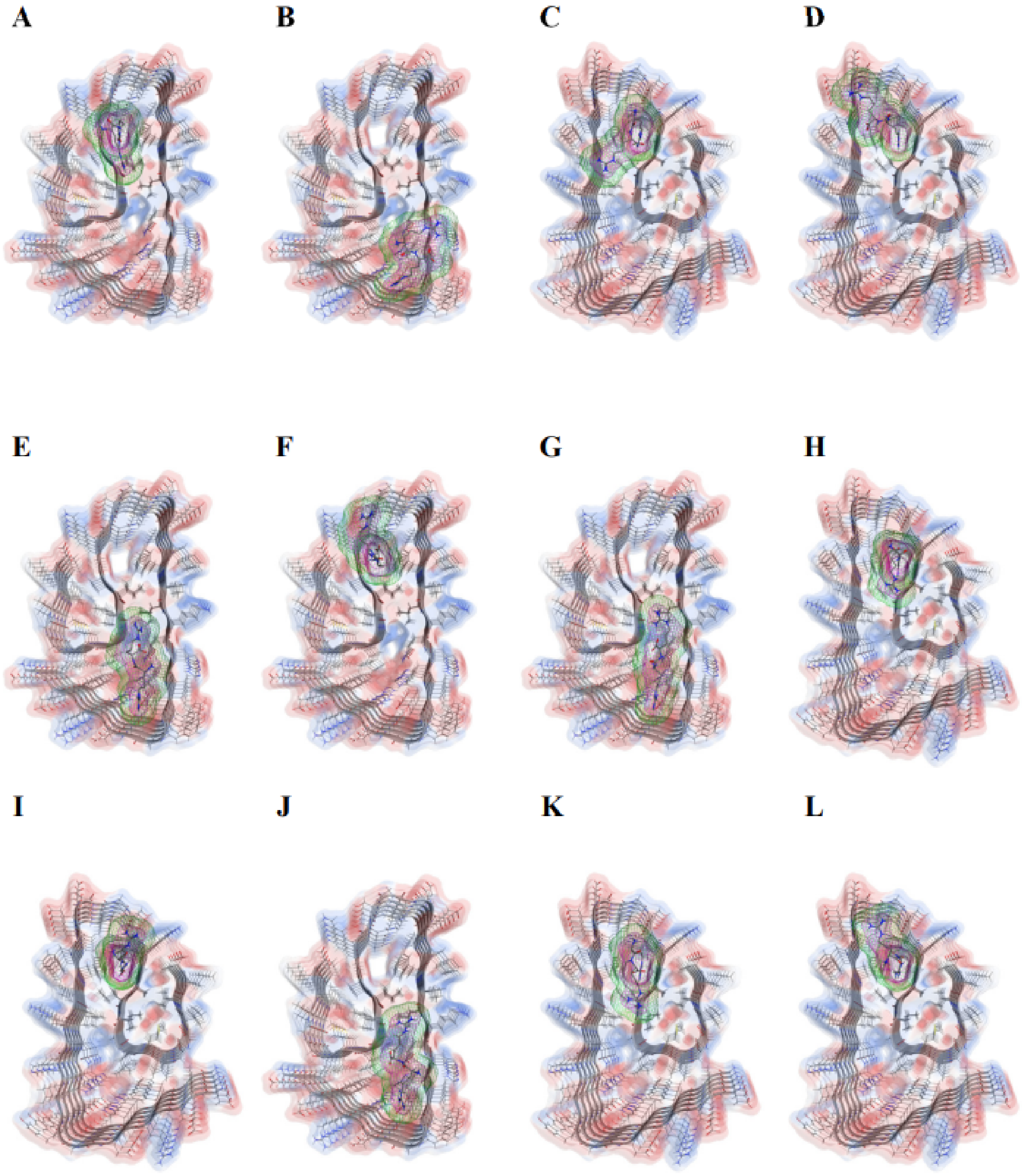

Supplementary Figure S3. Docking position of arginine dipeptide (RR) on Aβ_{42} pentamer. The conformation of Aβ_{42} pentamer was adapted from the structure (PDB #5oqv) determined by cryo-electron microscopy. **A–L.** Binding sites and conformation of RR on Aβ_{42}. The magenta mesh represents molecular surface of RR, and the green mesh represents the boundary of van der Waal's force.
